# Age and Sex Interact to Mediate the Effects of Intermittent, High-Dose Ethanol Exposure on Behavioral Flexibility

**DOI:** 10.3389/fphar.2017.00450

**Published:** 2017-07-07

**Authors:** Jacqueline M. Barker, Kathleen G. Bryant, Jennifer I. Osborne, L. J. Chandler

**Affiliations:** Department of Neuroscience, Medical University of South Carolina, CharlestonSC, United States

**Keywords:** adolescence, habit, behavioral flexibility, ethanol exposure, rat models, sex differences

## Abstract

Human alcoholics have been shown to have impaired cognitive control over actions and increased reliance on habitual response strategies. While it is unclear in humans whether these differences predate ethanol exposure or result from chronic drinking, data from animal studies suggest that ethanol acts to promote the development of inflexible behaviors. Here, we investigated how intermittent exposure to high doses of ethanol impacts the ability to flexibly regulate behavior in a habit model. As adolescence, may represent a period of increased drug taking and developmental vulnerability that may impact adult behavior, we compared the effects of high-dose ethanol exposure during adolescence to exposure during adulthood in male and female rats. Our findings indicated that the effects of intermittent, high-dose ethanol exposure on habitual behavior is mediated by age and sex such that ethanol exposure during adolescence promoted the use of habitual response strategies in adult females, but not males, and that the opposite pattern emerged following intermittent, high-dose ethanol exposure in adult rats.

## Introduction

The development of alcohol use disorders is associated with a reduction in the ability to flexibly regulate behaviors. The ability to balance efficiency and flexibility in order to alter and adapt behavior is a critical function in changing environments. Shifting between cognitive regulation of behavior and inflexible stimulus-driven behavior can be modeled by the transition between goal-directed and habitual response strategies. When a new behavior is learned, it is typically performed in a goal-directed fashion. That is, behavior is performed in relationship to its outcome. After time and repeated performance, a transition to habitual response strategies may occur in which behavior is no longer mediated by its outcome.

Inflexible behavior is implicated in a number of neuropsychiatric illnesses characterized by deficits in cognitive control, such as addiction and obsessive compulsive disorder. Indeed, data from human neuroimaging studies revealed impairments in behavioral flexibility and an overreliance on habitual response strategies in abstinent alcoholics (Sjoerds et al., 2013). Though deficits in cognitive control are a hallmark of addictive disorders, it is unclear whether this phenotype is the result of chronic ethanol exposure in humans, or rather is a risk factor predating the development of alcohol use disorders. Evidence from animal models has suggested that innate individual differences in cue reactivity and incentive motivation can predict subsequent differences in drug self-administration (Flagel et al., 2009; Saunders and Robinson, 2013) as well as the development of inflexible behaviors (Barker et al., 2012, 2014). In addition, sex differences in the propensity to develop habits have been reported such that while female rodents develop sucrose-seeking habits more quickly than males (Quinn et al., 2007), male rodents show more rapid development of habitual ethanol seeking (Barker et al., 2010). It is possible that sex differences in habit formation relate to differences in motivation to consume ethanol or differences in ethanol reinforcement. While sex differences in ethanol consumption have been observed, females generally self-administer ethanol at higher rates than males (e.g., Barker et al., 2010). It is alternatively possible that sub-chronic ethanol exposure differentially impacts loss of cognitive control over actions in males versus females. Indeed, while a growing body of literature indicates that ethanol exposure itself may act to promote the development of inflexible behaviors, including habits (Corbit et al., 2012), the bulk of this research has been performed in males.

In humans, the onset of drinking behavior during adolescence is highly predictive of subsequent development of alcohol use disorders, and as such, adolescence may mark a period of unique vulnerability to the effects of alcohol on cognitive control. Adolescence is characterized by increased risk-taking (Laviola et al., 2003) and goal-seeking behavior (Doremus-Fitzwater and Spear, 2016), and adolescent males are resistant to the development of habitual ethanol seeking (Serlin and Torregrossa, 2014). Binge-like ethanol exposure during adolescence has been shown to have long-lasting effects on behavior, including impairments in fear learning and extinction (Broadwater and Spear, 2013) and in set-shifting (Gass et al., 2014). These findings suggest that the impact of ethanol exposure on the development of habitual reward seeking may not only be sex-specific, but may also depend upon the developmental age when exposure occurs. Here, we investigated how intermittent, high-dose ethanol exposure during either adolescence or adulthood impacted the development of habitual reward seeking in male and female rats.

## Materials and Methods

### Animals

Male and female Long Evans rats (Harlan Labs) were delivered to the Medical University of South Carolina animal facility at either postnatal day (PND) 22 (adolescent ethanol exposure) or 60 (adult ethanol exposure). Rats were housed on a reverse light cycle with 12 h light:12 h dark. All behavioral procedures occurred during the dark cycle. Adolescent rats were housed with litter-mates of the same sex, and adult rats were pair housed unless separation was required because of injury or fighting. All procedures were conducted with approval of the Medical University of South Carolina Institutional Animal Care and Use Committee.

### Intermittent Ethanol Exposure Procedure

**FIGURE 1:**
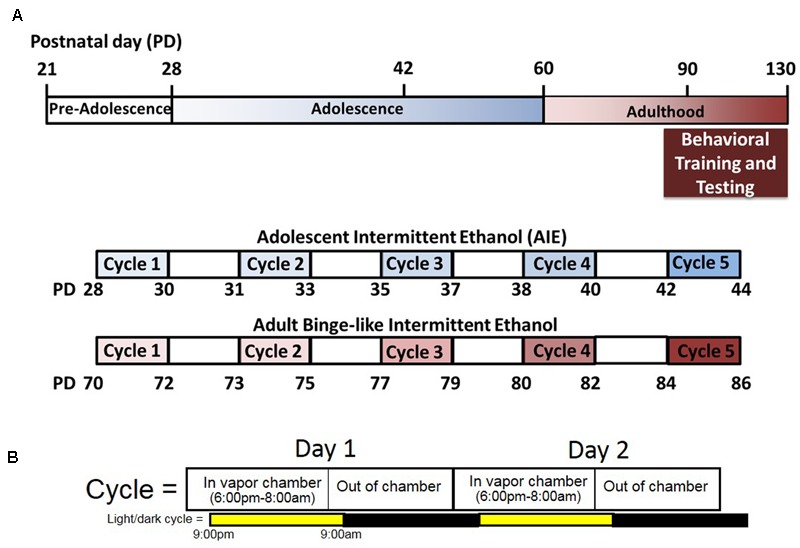
Experimental design and time-line. **(A)** Male and female rats were exposed to intermittent ethanol exposure during either adolescence (blue) or adulthood (red). All rats underwent five cycles of exposure to ethanol. For adolescent ethanol exposed rats, exposure occurred between PND 28–44 and testing occurred after PND 80. For adult exposed rats, exposure occurred between PND 70–86 and testing occurred after PND 120. **(B)** Each cycle of ethanol exposure consisted of two 14-h exposures that began 3 h prior to the start of the light cycle and ended 1 h prior to the start of the dark cycle.

**FIGURE 2:**
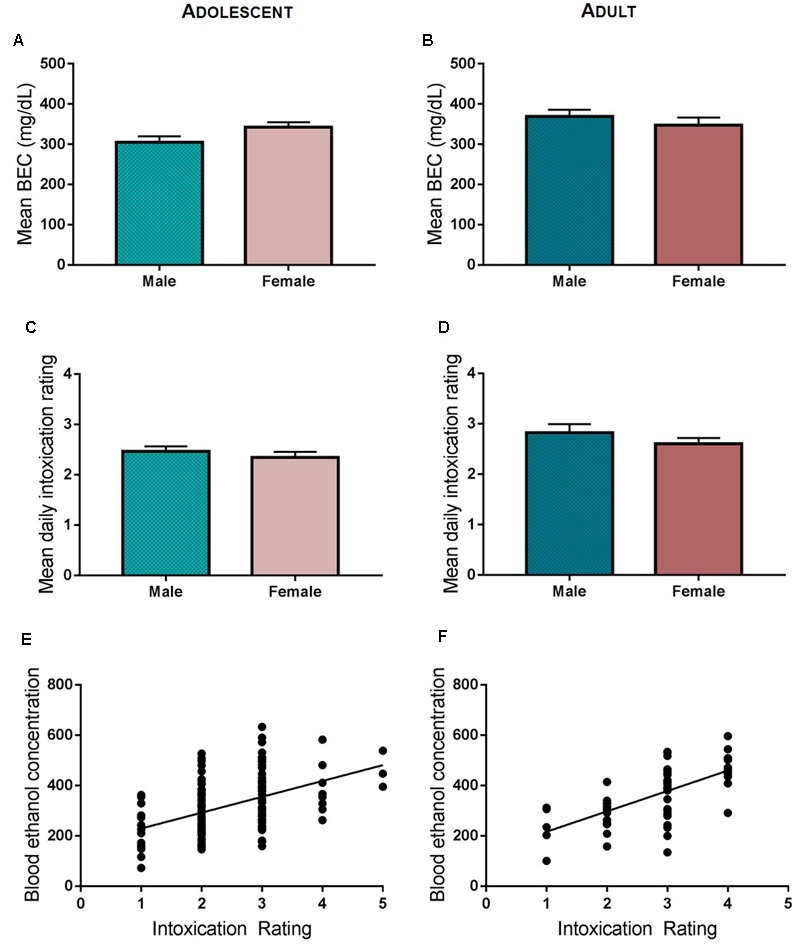
Ethanol vapor exposure. Mean intoxication ratings were comparable for adolescent **(A)** and adult **(B)** ethanol exposed rats and did not differ between male and female rats. Male and female rats had similar blood ethanol levels during adolescent **(C)** or adult **(D)** ethanol vapor exposure. BECs and intoxication ratings were significantly correlated in both adolescent exposed **(E)** and adult exposed rats **(F)**.

Adolescent and adult rats assigned to undergo ethanol exposure were subjected to five cycles of ethanol exposure via vapor inhalation (**Figure 1A**). Control rats were moved to the chamber exposure room each day but did not undergo vapor inhalation of ethanol. Each cycle of ethanol exposure consisted of two consecutive episodes of exposure, with each exposure lasting 14 h in the vapor chambers (**Figure 1B**). Vapor chamber exposure began 3 h prior to the onset of the light cycle, and rats are removed from vapor chambers 1 h prior to the onset of the dark cycle at which time intoxication ratings and tail-vein blood samples are obtained. Adolescent ethanol exposure spanned the period from early to mid- adolescence (PND 28–44) and involved exposure to ethanol vapor on PND 28 and 29 (Cycle I), 31 and 32 (Cycle II), 35 and 36 (Cycle III), 38 and 39 (Cycle IV), and 42 and 43 (Cycle V). Following ethanol exposure, rats were returned to the colony where they were maintained on *ad libitum* food and water until at least PND 80. To investigate the effects of intermittent, high-dose ethanol exposure during adulthood, exposure began at PND 70 and rats were exposed to ethanol vapor on PND 70 and 71 (Cycle I), 73 and 74 (Cycle II), 77 and 78 (Cycle III), 80 and 81 (Cycle IV), and 84 and 85 (Cycle V). Rats that underwent intermittent, high-dose ethanol exposure in adulthood were maintained on *ad libitum* food and water for 36 days prior to any behavioral testing in order to match the time between adolescent ethanol exposure and assessment of the development of habitual reward seeking in adulthood. Tail vein blood was collected on a subset of ethanol vapor exposure sessions for both adolescent and adult-exposed animals in order to measure blood ethanol concentration (BEC), and the level of intoxication was determined at the end of each vapor chamber exposure using a previously described 5-point behavioral intoxication rating scale (Nixon and Crews, 2002; Gass et al., 2014) as follows: 1 = not intoxicated (no apparent signs of intoxication); 2 = low intoxication (slight motor impairment); 3 = moderate intoxication (obvious motor impairment, but able to walk); 4 = high intoxication (loss of righting reflex for >30 s, dragging of abdomen when walking, severe motor impairment, maintains eye blink reflex); 5 = extreme intoxication (loss of righting reflex, loss of eye blink reflex, may be unconscious, or difficult to arouse). We targeted intoxication ratings between 2 and 3 (low to moderate intoxication levels; **Figures 2C,D**). Targeted BECs between 250 and 350 mg/dL and low to moderate intoxication ratings were chosen to model ethanol consumption patterns in young adults drinkers. Recent reports suggest that in natural environments (i.e., at a bar) adolescent and young adults achieve estimated blood alcohol concentrations within this range (Treloar et al., 2017). Intoxication ratings have been shown previously to correlate with BEC, and indeed our findings revealed that intoxication ratings in both adolescent and adult ethanol exposed animals significantly correlated with BECs (**Figures 2E,F**). Since obtaining tail vein blood from unintoxicated rats is a much more stressful procedure than it is with intoxicated rats, blood was not obtained from control rats so as not to introduce this stress as an experimental confound.

### Behavioral Training and Testing

After at least 36 days from the last cycle of ethanol exposure, rats were food restricted to approximately 90% of their free feeding weights and then trained to self-administer 10% sucrose via two levers in a standard rat operant chamber from Med-Associates (St. Albans, VT, United States). Operant chambers were housed within a sound-attenuating box. Each chamber was equipped with a 28 V house light and had two retractable levers on the left and right panels of the right wall. A magazine was located at the bottom of the middle panel on the right side of the wall. Sucrose was delivered to a well in the magazine via a syringe pump housed within the sound attenuating chamber but outside of the operant box. A fan provided background noise and ventilation. No additional tones or cues were presented.

Each day, rats had a 30-min training session during which each lever was presented separately. Each lever was only available for 15 min and the order of lever availability was alternated daily. A press on either lever was initially reinforced on a fixed ratio 1 (FR1) schedule in which each lever press resulted in a single delivery of sucrose. After stable responding (≥ to 3 consecutive days) of at least 30 presses per lever, reinforcement schedules were transitioned to either an action-promoting random ratio (RR) 5 or a habit-promoting random interval (RI) 30 schedule (Adams and Dickinson, 1981; Gremel and Costa, 2013). The use of these schedules enabled within-subjects assessment of response strategy under conditions where either goal-directed actions (RR schedule) or habitual response strategies were expected (RI schedule). On the RR5 schedule, the number of lever presses controlled reinforcer delivery. On average, the fifth response was reinforced, but the program software randomly generated the actual response requirement. On the RI30 schedule, the first lever pressed after a randomly determined interval (averaging 30 s) had elapsed was reinforced. Unlike the RR schedule, reinforcer delivery on the RI schedule was not related to the number of lever presses. The response schedule assigned to each lever was consistent across training for each animal (i.e., left lever was always the ‘interval’ lever and right lever was always the ‘ratio’ lever). After two sessions on the RR5/RI30 schedules, these were transitioned to RR10 and RI60 schedules for an additional eight sessions.

Following eight sessions on the RR10/RI60 schedule, response strategy was assessed using a specific satiety outcome devaluation procedure. Because habits are by definition insensitive to changes in outcome value, devaluation procedures enable the determination of whether rats are relying on habitual response strategies in which responding is unaffected by devaluation, or are using goal-directed strategies in which reward seeking behavior is reduced following devaluation. Rats underwent both a ‘non-devalued’ and ‘devalued’ testing procedure for the RR and RI levers. During the ‘devalued’ condition, rats received access to a 10% sucrose bottle for 1 h prior to a 10 min test session in a novel cage. During these test sessions, conditions were identical to training except that they were performed in extinction in which lever presses did not result in reinforcer delivery. Following this test session, rats were again provided 1-h access to a 10% sucrose bottle to confirm devaluation. In the ‘non-devalued’ condition, rats received 1-h access to home cage chow prior to testing. Between all devaluation tests, rats had 1 additional training session on the RR10/RI60 schedule and the order of testing was counterbalanced.

### Statistical Analyses

Analysis was performed in SPSS using repeated measures ANOVA and *t*-tests as appropriate. *Post hoc t*-test comparisons were Bonferroni corrected.

## Results

### Intoxication Ratings

Comparison of daily intoxication ratings with BEC values across groups revealed no difference between male and females, or between adult and adolescent rats. Data were collapsed across exposure cycles for analysis. Neither mean BECs nor intoxication ratings significantly differed between males and females in adolescent rats (*t* = 0.1828, *p* > 0.5; *t* = 0.755, *p* > 0.4; **Figures 2A,C**) or in adult rats (*t* = 0.876, *p* > 0.3; *t* = 1.084, *p* > 0.2, respectively; **Figures 2B,D**). Intoxication ratings and BECs were positively correlated on days that BEC’s were taken, indicating that intoxication ratings reliably reflect BEC (adolescent ethanol exposure, Pearson’s correlation, *r*^2^ = 0.208, *p* < 0.001; adult ethanol exposure, Pearson’s correlation, *r*^2^ = 0.407; *p* < 0.001; **Figures 2E,F**).

### Acquisition of Sucrose Seeking

#### Adolescent Ethanol Exposure

**FIGURE 3:**
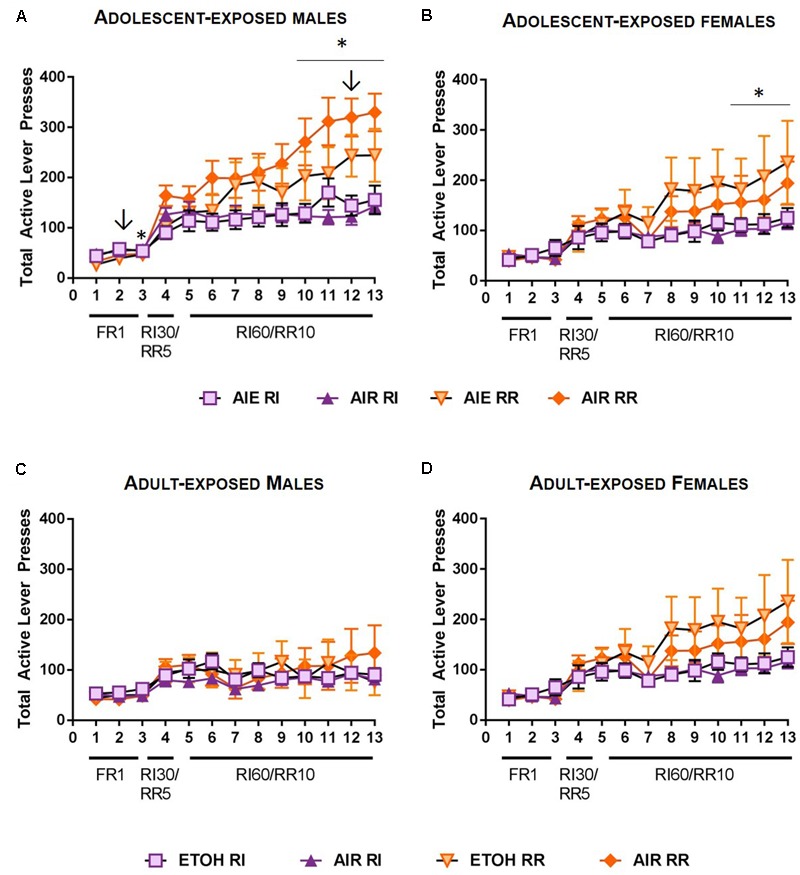
Acquisition of sucrose seeking in rats exposed to ethanol. **(A)** In adult male rats that had been subject to adolescent intermittent ethanol (AIE), responding on the RR lever was significantly higher than responding on the RI lever on days 2 and 12 (arrows). In control (AIR) male rats, responding was significantly higher on the RR lever on days 3, and 10–13. **(B)** In adult female rats that had been subject to AIE, no differences in response rates were observed between AIE and AIR exposed rats. Responding was significantly higher on the RR lever than the RI lever on the final 3 days or RI60/RR10 responding for both groups. **(C)** In male rats subjected to intermittent ethanol (ETOH) exposure in adulthood, no differences in responding between ethanol-exposed and control rats were observed, nor did adult ethanol exposure alter response rates on the RR and RI levers. **(D)** In female rats exposed to ethanol in adulthood, no significant differences in responding on RR versus RI levers were observed. ↓*p* < 0.05; ^∗^*p* < 0.05.

A rmANOVA on total lever presses across acquisition indicated main effects of reinforcement schedule [*F*(1,37) = 26.233, *p* < 0.001] and day of training [*F*(12,444) = 50.527, *p* < 0.001]. In addition, significant interactions between schedule, day of training, and sex were observed [*F*(12, 444) = 2.427, *p* < 0.01], as well as schedule x day x ethanol exposure [*F*(12,444) = 3.104, *p* < 0.001]. Follow-up rmANOVA indicated that in male rats, reinforcement schedule, ethanol exposure, and day of training interact to moderate responding [*F*(12,228) = 2.656, *p* < 0.01]. *Post hoc* comparisons further indicated that in air control males, responding on the RR vs RI levers was significantly different on days 3, 10, 11, 12, and 13 of training (*p*-values < 0.05; **Figure 3A**). In contrast, in ethanol exposed rats, responding differed on RR and RI levers only on day 2 and 12 of training (*p*-values < 0.05). In females, ethanol exposure did not impact responding during acquisition [main effect *p* > 0.2, no significant interactions], but a schedule by day interaction was present [*F*(12, 216) = 5.070, *p* < 0.001]. Follow-up comparisons indicated differences in responses on the RI vs RR lever on days 11, 12, and 13 for female rats (*p* < 0.05; **Figure 3B**).

#### Adult Ethanol Exposure

A rmANOVA on total lever presses across acquisition indicated a main effect of schedule on responding [*F*(1,26) = 4.723, *p* < 0.05] and a main effect of day of training [*F*(12,312) = 17.437, *p* < 0.001], consistent with an increase in responding across training. In addition, a significant day x sex interaction was observed [*F*(12, 312) = 2.843, *p* < 0.01] as well as a reinforcement schedule x day of training interaction [*F*(12,312) = 3.102, *p* < 0.001]. No significant main effect of ethanol exposure was observed [*F*(1,26) = 0.243, *p* > 0.6], and no significant ethanol exposure interactions were observed with any other measures (*p*-values > 0.4). In male rats, no main effect of schedule [*F*(1,12) = 0.505, *p* > 0.4] or schedule × day interaction were observed [*F*(12,180) = 0.605, *p* > 0.8] indicating comparable levels of responding on the RR and RI schedules. However, a main effect of day was observed [*F*(12,180) = 5.529, *p* < 0.001] consistent with increased responding across training (**Figure 3C**). In female rats, in addition to a significant effect of day [*F*(12,156) = 12.724, *p* < 0.001], a significant schedule × day interaction was observed [*F*(12,156) = 3.4, *p* < 0.001], indicating that response rates were distinct on RI and RR schedules. While a main effect of schedule [*F*(1,156) = 4.591, *p* = 0.05] is consistent with a higher response rate on the RR schedule than the RI schedule, post-hoc comparisons did not indicate significant differences in responding on the RR versus RI schedule on individual days of training (**Figure 3D**).

### Adolescent Ethanol Exposure Promoted Used of Habitual Response Strategies in Females

**FIGURE 4:**
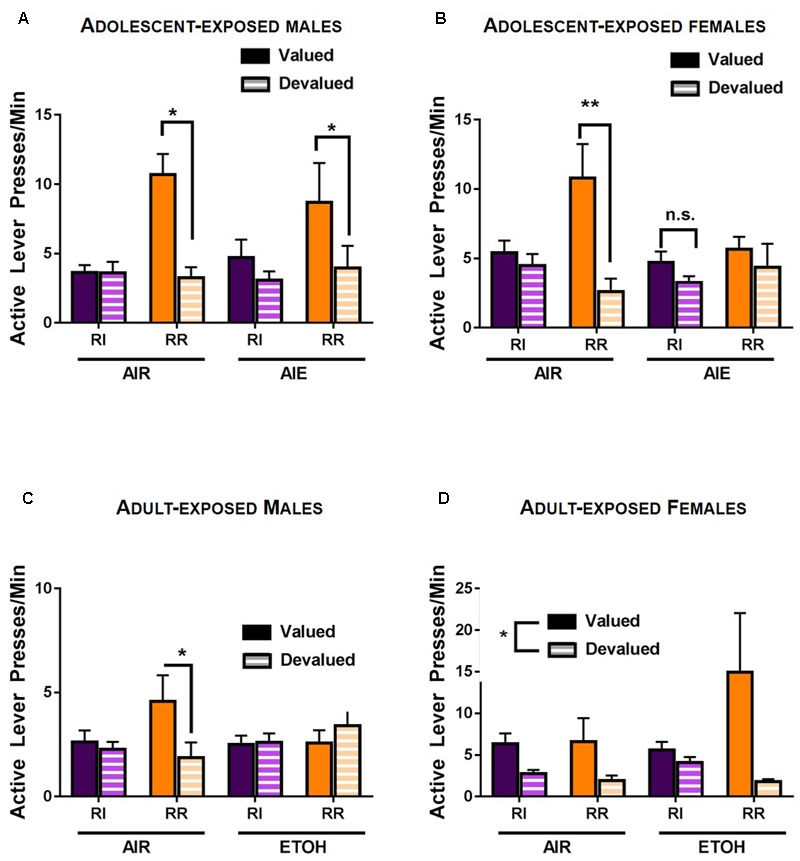
Response strategy selection in an outcome devaluation test in adult rats following ethanol exposure. **(A)** In adult male rats, responding on the RI habit-promoting lever was habitual in both air control and AIE exposed groups, as evidenced by a lack of reduction in response rate when the outcome was devalued by specific satiety. In contrast, responding on the RR lever was goal-directed (sensitive to outcome devaluation) in male rats in both the control (AIR) and ethanol exposed groups. **(B)** AIR exposed female rats showed similar patterns of responding to males. In control female rats, responding on the RI schedule was habitual (i.e., was insensitive to outcome devaluation), while responding on the RR lever was goal-directed and sensitive to outcome devaluation. In contrast, AIE exposed females showed habitual response strategies on both the RI lever and the RR lever; responding on both the RR and RI levers was insensitive to outcome devaluation. **(C)** Air exposed male rats were insensitive to outcome devaluation on the RI lever, consistent with habitual behavior, but were sensitive to outcome devaluation on the RR lever, indicative of goal-directed actions. In contrast, adult ETOH exposed males were insensitive to outcome devaluation on both levers, indicative of habitual response strategies during responding in both action- and habit-promoting conditions. **(D)** In female rats, a main effect of devaluation was present, indicating lower response rates during devaluation in all conditions. This was not mediated by schedule or adult ethanol exposure. ^∗^*p* < 0.05.

For adult rats exposed to alcohol during adolescence, a rmANOVA of response rates during the outcome devaluation test indicated a reinforcement schedule × devaluation × ethanol exposure interaction [*F*(1,41) = 5.621, *p* < 0.05] as well as a significant schedule × devaluation interaction [*F*(1,41) = 9.791, *p* < 0.01]. Main effects of reinforcement schedule [*F*(1,41) = 11.947, *p* < 0.01] and devaluation [*F*(1,41) = 27.303, *p* < 0.001] were also observed. Follow-up comparisons indicated that in males, no main effect of ethanol exposure [*F*(1,21) = 0.035, *p* > 0.8] or interactions with ethanol exposure were observed (*p*-values > 0.2). A significant schedule × devaluation was observed [*F*(1,21) = 7.928, *p* < 0.01], as well as main effects of schedule [*F*(1,21) = 8.407, *p* < 0.01] and devaluation [*F*(1,21) = 15.417, *p* < 0.001]. *Post hoc* analyses indicated no difference between response rates on the RI lever during valued versus devalued conditions (*p* > 0.3), consistent with habitual behavior. In contrast, response rates on the RR lever were lower during the devalued test than the valued test (*p* < 0.01; **Figure 4A**), consistent with goal-directed behavior. In female rats, a schedule × devaluation × ethanol exposure interaction was indicated by rmANOVA [*F*(1,20) = 4.709, *p* < 0.05], as well as a devaluation × ethanol exposure interaction [*F*(1,20) = 2.37, *p* < 0.05). In addition, a significant main effect of devaluation was observed [*F*(1,20) = 12.013, *p* < 0.01]. Subsequent comparisons indicated that adolescent air exposed female rats exhibited habitual responding on the RI lever and did not change response rates when the outcome was devalued (*p* > 0.4). In contrast, air exposed females exhibited goal-directed reward seeking on the RR lever and lower response rates on the RR lever (*p* < 0.05). Female rats that had been subjected to intermittent, high-dose ethanol exposure during adolescence, however, showed habitual behavior on both the RR and RI levers (*p* > 0.8 and *p* > 0.1, respectively), consistent with increased reliance on habitual response strategies in female rats following adolescent ethanol exposure (**Figure 4B**).

### Adult Ethanol Exposure Promoted the Use of Habitual Response Strategies in Males

For rats exposed to ethanol during adulthood, a rmANOVA of response rates during the outcome devaluation tests indicated a four-way interaction between schedule × sex × ethanol exposure × devaluation [*F*(1,26) = 4.276, *p* < 0.05], as well as a main effect of schedule [*F*(1,26) = 13.378, *p* < 0.01], a schedule × sex interaction [*F*(1,26) = 9.216, *p* < 0.01] and a devaluation × schedule interaction [*F*(1,26) = 4.725, *p* < 0.05]. No main effect of devaluation was present [*F*(1,26) = 1.78, *p* = 0.194] or main effect of ethanol exposure [*F*(1,26) = 1.172, *p* = 0.289]. There was however, a main effect of sex on response rates [*F*(1,26) = 7.566, *p* < 0.05].

To deconstruct significant interactions, rmANOVAs were performed separately on response rates for male and female rats. In male rats, this analysis revealed a significant interaction of schedule × devaluation × ethanol exposure [*F*(1,14) = 7.349, *p* < 0.05] and a schedule × ethanol interaction [*F*(1,14) = 5.847, *p* < 0.05]. *Post hoc* analysis indicated no significant effect of devaluation on responding on the RI schedule for ethanol exposed (*p* > 0.8) or air exposed males (*p* > 0.4). In contrast, air exposed control males showed lower rates of responding on the devalued lever in the RR schedule (*p* < 0.5) (**Figure 4C**), indicative of goal-directed behavior. In adult ethanol exposed males, however, response rates did not differ between the valued and devalued conditions on the RR schedule (*p* > 0.2), indicative of habitual responding on the ‘action-promoting’ lever. In female rats, rmANOVA indicated a main effect of devaluation [*F*(1,12) = 10.295, *p* < 0.01), and a non-significant trend toward a devaluation x schedule interaction [*F*(1,12) = 3.299, *p* = 0.09) (**Figure 4D**), suggesting that ethanol exposure in adult females did not impact response strategy selection.

## Discussion

Our findings indicate that age and sex interact to determine the effects of intermittent, high-dose ethanol exposure on habitual behavior. In particular, we found that intermittent ethanol exposure during adolescence promoted the reliance on habitual response strategies in female rats, while response strategy was not impacted in males. In contrast, exposure to ethanol in adulthood using an identical exposure paradigm was associated with facilitation of habit formation in males but not females. These findings suggest that while intermittent high-dose ethanol exposure altered the ability to flexibly regulate behavior, the effect is dependent upon the age of ethanol exposure as well as sex.

In the adult exposure paradigm, air exposed control males exhibited habitual reward seeking when responding on an interval (habit promoting) schedule, but retained sensitivity to outcome devaluation on a ratio (action promoting) schedule. In males that were exposed to ethanol during adulthood, however, outcome devaluation had no impact on responding on either the RI or the RR schedule, indicating a greater propensity toward habitual response strategies. Female rats showed no effect of adult ethanol exposure on response strategy selection. Given previous reports that adult males develop ethanol seeking habits more rapidly than sucrose seeking habits (Dickinson et al., 2002), and that males form ethanol seeking habits more quickly than females (Barker et al., 2010), these data suggest that in adults, intermittent ethanol exposure may act to promote habitual response strategies in males but not females.

As with investigation of the consequences of adolescent ethanol exposure in females, there is a paucity of data describing sex differences in the impact of intermittent ethanol exposure in adult animals. Current findings suggest that males and females may be differentially sensitive to the both the aversive and rewarding effects of ethanol exposure. For example, while both adult males and females exhibit increases in anxiety following a bout of intoxication, this effect is exaggerated in adult males (Varlinskaya and Spear, 2004). This study also observed protracted elevation in BECs in adult males as compared to adult females, and that this effect was absent in adolescents (Varlinskaya and Spear, 2004). Together with additional literature demonstrating more rapid recovery from early withdrawal in females than in males (Devaud and Chadda, 2001), these findings suggest that adult males may be differentially sensitive to repeated bouts of intoxication and withdrawal than females. Consistent with these sex differences, male rats show persistent elevations in corticosterone following ethanol withdrawal, while corticosterone levels return to baseline in females (Alele and Devaud, 2006). Because chronic corticosterone itself can facilitate reliance on habitual response strategies in males (Gourley et al., 2012; Swanson et al., 2013), it is possible that repeated ethanol intoxication and withdrawal in adult males facilitates habitual behavior via its effects on the glucocorticoid system.

Interestingly, adolescent ethanol exposed male rats did not differ in response strategy selection compared to air exposed controls when tested in adulthood. Both ethanol-exposed and air control males showed habitual response strategies on the interval schedule and goal-directed actions when responding on the ratio schedule. Adolescent males have been shown to be resistant to habitual ethanol seeking relative to adult males (Serlin and Torregrossa, 2014), which is consistent with increased goal-oriented behavior in adolescent males (Doremus-Fitzwater and Spear, 2016). This suggests that ethanol exposure may not act on neurocircuitry to facilitate habit formation and expression in adolescent males in the same way it does in adults (Dickinson et al., 2002; Barker et al., 2010; Corbit et al., 2012). While a number of groups have identified protracted adolescent-like phenotypes following binge-like and intermittent ethanol exposure, our findings were not consistent with this in regard to response strategy selection. We observed that adult males were capable of habit formation following adolescent ethanol exposure – i.e., male rats showed habitual responding under habit-promoting conditions - and thus did not exhibit an adolescent-like resistance to habit formation.

In contrast to our observations in male rats, adolescent ethanol-exposed female rats demonstrated facilitated habitual reward seeking in adulthood. The precise timing of ethanol exposure during adolescence has been shown to have distinct effects on adult behavior (Spear, 2015). In particular, ethanol exposure in early adolescence that encompasses the onset of puberty may have greater effects on adult behavior than ethanol exposure restricted to later adolescence (Spear, 2015). It is possible that the sex differences we observed result from differences in pubertal onset – females mature more rapidly than males and undergo puberty earlier than male rats. Because our ethanol vapor procedure is restricted to early- to mid- adolescence (i.e., is ended at PND 44), this period encompasses the pubertal maturation period of female rats, but may not cover the entire pubertal period of male rats. The timing of drug alcohol exposure relative to puberty may mediate the effect of exposure on neuronal maturation. For example, the effects of amphetamine withdrawal on dopamine receptor expression in the medial prefrontal cortex (mPFC) was dependent upon the timing of drug exposure such that reductions in dopamine D1 receptors in mPFC occurred only in animals exposed to amphetamine prior to pubertal onset (Kang et al., 2016). Recent studies from our lab also suggest that intermittent ethanol exposure during this time period of development in males resulted in profound changes in dopamine neurotransmission in the prelimbic cortex, including reductions in both D1 receptor expression and expression of COMT, which together with the norepinephrine transporter, is the primary mediator of the extracellular levels of dopamine in the PFC (Trantham-Davidson et al., 2017). It is important to note that to date, investigation of dopamine system alterations following intermittent ethanol exposure in this model is largely limited tomales, and the effects of adolescent ethanol exposure on mPFC dopamine neurotransmission in females is poorly understood. Prefrontal dopamine signaling regulates behavioral flexibility and cognitive control in a number of different tasks including impairments in the ability to detect change in reward value (Winter et al., 2009) and set shifting (Floresco et al., 2006). It has also previously been reported that mPFC dopamine signaling can acutely and bidirectionally regulate habitual reward seeking (Barker et al., 2013). Importantly, changes in dopamine signaling following intermittent ethanol exposure are long lasting. Though findings on the role of PFC dopamine signaling specifically are lacking, chronic changes in dopamine following chronic cocaine (Halbout et al., 2016) or amphetamine (Nelson and Killcross, 2006) are known to promote reliance on habit-like response strategies that are PFC-dependent (Nelson and Killcross, 2013).

In addition to changes in prefrontal dopamine signaling, intermittent ethanol exposure during adolescence has been shown to disrupt GABAergic neurotransmission in the PFC in both male and female adult rats (Centanni et al., 2017; Trantham-Davidson et al., 2017), including impairments in tonic GABA_A_ currents (Centanni et al., 2017). Normal maturation of the mPFC GABA system has been shown to be required for appropriate goal-directed behaviors in adulthood (Butkovich et al., 2015), suggesting that alterations in GABA_A_ signaling following adolescent alcohol exposure could contribute to the alterations in response strategy selection in adulthood observed in the present study. However, the absence of sex differences in tonic GABA current in the Centanni et al. (1999) study suggests that dysregulation of GABAergic transmission in the PFC following adolescent ethanol exposure is unlikely to explain the increased reliance on habitual response strategies in female rats. Interestingly, sex-specific effects of adult ethanol exposure on GABA_A_ receptor subunit expression have been reported (Devaud et al., 1999). Female ethanol withdrawn rats show inceased sensitivity to neuroactive steroids thought to act at.

## Summary and Implications

The ability to toggle between actions and habits is critical to maximize behavioral efficiency. Our findings indicate that intermittent, high-dose ethanol exposure impacts the ability to flexibly regulate behavior in a sex- and age- dependent manner. We observed that high-dose ethanol exposure during adolescence promoted reliance on habitual response strategy in adult female but not male rats. In contrast, this same intermittent, high-dose ethanol exposure paradigm in adults facilitated the use of habits in male but not female rats. Together, these findings point to a need for an increased understanding of sex differences in the impact of drugs and alcohol on the neurocircuits that mediate cognitive control and behavioral flexibility, and how restricted access to drugs and alcohol may have long-lasting effects on the plasticity and function of these circuits.

## Author Contributions

JB, JO, KB performed the experiments. JB and LC designed the experiments and wrote the manuscript.

## Conflict of Interest Statement

The authors declare that the research was conducted in the absence of any commercial or financial relationships that could be construed as a potential conflict of interest.

The reviewer KS and handling Editor declared their shared affiliation, and the handling Editor states that the process nevertheless met the standards of a fair and objective review.
